# Cloning and expression of natively paired antibody fragments from single B cells of immunized rhesus macaques

**DOI:** 10.3389/fimmu.2026.1832934

**Published:** 2026-06-16

**Authors:** Johid Reza Malik, Courtney V. Fletcher, Sean N. Avedissian

**Affiliations:** Antiviral Pharmacology Laboratory, College of Pharmacy, University of Nebraska Medical Center, Omaha, NE, United States

**Keywords:** FAB, FHBP, mAb, protein expression, single B-cell, immunization

## Abstract

Antigen-binding fragments (Fabs) are emerging biochemical tools for treating and managing several diseases and conditions. Though full-length monoclonal antibodies (mAbs) have great potential, their higher molecular size, time required for generation, and cost remain major limiting factors. Fabs, being smaller and having similar affinity to mAbs for their target, can reach the affected site easily, where penetration of mAb might be hindered. Additionally, with fewer amino acids than mAbs, Fabs can be genetically engineered for the required function. In the present work, we have used a combination of state-of-the-art technologies and classical methods for Fab generation from a single B cell of rhesus macaque previously immunized with factor H binding protein (FHbp) of *Neisseria meningitidis* (Nm). FHbp was selected for its potency as an immunogen and importance in vaccine development against Nm. Using conjugated FHbp as a bait to sort single B cells, expressing antibodies against FHbp increases the probability of finding antigen-specific B cells. We have not only developed a protocol for Fab generation but also provided proof of concept for increasing the yield of toxic or poorly expressed proteins in bacteria by the inclusion of ethanol in bacterial culture.

## Introduction

Fabs (antibody fragments) are specificity-directing functional parts of antibodies (Abs) and provide a better platform for manipulating target specificity or affinity along with efficacy, compared to Abs (1). The yeast surface display (YSD) method has been used for affinity modulation and expression of Abs (2), (3). However, the time required to find a suitable antibody, associated labor, and cost are limitations (4). Single and specific B-cell sorting based on antigen–antibody detection by flow cytometry and the downstream reverse transcription-polymerase chain reaction (RT-PCR) technology for molecular cloning of Fabs are increasingly gaining attention because of their low cost but high-throughput and efficient native VH–VL pairing. Moreover, a native pairing of an immunoglobulin heavy chain (VH) and a light chain (VL) is essential for a potential candidate antibody to be functional (5, 6). The time required to generate mAbs and the cost associated with identifying them are a major concern, which reflects on the high commercial value of such mAbs (7). Despite being an efficient and target-oriented drug formulation, antibody-based molecules still need fine-tuned engineering given their larger molecular size (8, 9). Consequently, antigen binding Fabs have become a better treatment option than mAbs because of their smaller size (10) and their tendency to be cleared at a faster rate from systemic circulation, compared to intact Abs due to their susceptibility to proteolysis in the absence of the constant “fragment crystallizable” (Fc) region and glycosylation (11). Target specificity and the potential to treat diseases make Fabs and mAbs (monoclonal antibodies) better therapeutic agents and much-needed biochemical tools (12).

Additionally, the binding of Abs to surface receptors with high avidity can hinder the diffusion of unbound Abs to intracellular sites, suggesting local molecular crowding (13). Fab alleviates the size-related concern and provides a better platform for molecular engineering owing to its smaller molecular weight, though for therapeutic purposes, Fabs are promising only when the Fc region is not required for augmentation of other immune reactions like activation of the complement cascade. Fabs are progressively in demand for their specificity, smaller size, better stabilization, and ease of molecular engineering (14). Only limited studies have been published with an in-depth insight into Fab production along with characterization. These studies on Fab development employing single B-cell and RT-PCR technology used CD38 marker to select B cells instead of antigen-specific B cells. One such published study described a method for Fab DNA generation, but purification of Fab fragments was not attempted (15). A systematic and cost-effective approach is still required to develop libraries of Fabs from single-cell sorted B cells.

Pre-immunized samples for a specific antigen have an advantage in selecting a B-cell subset repertoire, which can be harvested to analyze Fab functionality (16, 17). Studies have shown that neutralizing Abs from rhesus macaque deliver better efficacy compared to the murine model (18). One study on immunized rhesus macaques concluded a higher number of B-cell lineages than earlier reports for humans or other non-human primates (19). Also, rhesus macaques are close to human physiology and cost-effective against the manufacturing and testing of defective or low immunogenicity products in humans (20). Factor H binding protein (FHbp) is one of the major antigens in the MenB-4C vaccine (Bexsero®) for the prevention of disease caused by *Neisseria meningitidis* and is the only antigen in the MenB-FHbp (Trumenba®) vaccine (21). Importantly, in all vaccines currently in use against *N. meningitidis* serogroup B, the major antigen, FHbp, shows very high affinity for human factor H (FH) (21). Based on earlier studies (22) and as part of a project to understand the differences in Ab repertoire elicited by wild-type (WT) and mutant FHbp, we used FHbp to conjugate Alexa-488. The macaques were immunized, as mentioned in earlier studies (23, 24), which have shown that a double mutant of FHbp (R41S and H248L) has a highly reduced affinity to bind FH and can act as a better immunogen (25). Accordingly, to rule out the possibility of FHbp binding with even a trace of FH already bound to the cell surface, we conjugated mutant FHbp to use it as bait to screen specific B cells for sorting by flow cytometry.

The expression of either Fab or full-length antibody in an *Escherichia coli* system is not as efficient as other protein production methods. The reason could be toxicity caused by Fab/Abs to *E. coli* (26), hindering protein production. To overcome the lower expression and alleviate the toxicity concern, several expensive expression vectors and various expression hosts available commercially are required, which add to the expression cost. Here, we have used a simple and universal lab reagent, ethanol, to achieve better expression of our Fabs in the *E. coli* system. Though not thoroughly exploited or investigated, using a lower percentage of alcohol can make a difference in the expression profile of some proteins (27), particularly for low-expressing proteins. Our data show that the Fabs that do not express well in bacterial fermentation systems can be expressed more highly with the addition of 3% ethanol. In summary, we describe a rapid and robust protocol for identifying antigen-specific B cells and a subsequent step-by-step methodology for expressing and purifying specific Fabs. Our protocol and data reveal that classical procedures and advanced technologies can be combined and optimized for the cost-effective production of desired Fabs.

## Materials and methods

### Isolation of B cells from FHbp-immunized macaque PBMCs by single-cell sorting

For our labs’ earlier project, mentioned in the Discussion section, Rhesus macaques were injected with either WT FHbp or double mutant FHbp (24). Throughout the study, the mutant refers to the double mutant of FHbp. Frozen macaque peripheral blood mononuclear cells (PBMCs) from monkey 120 injected with WT and another monkey (monkey 184) immunized with a double mutant of FHbp were used for single-cell sorting by flow cytometry. Blood collected from macaques immunized with WT or FHbp double mutant was subjected to Ficoll-based isolation and purification of PBMCs. Isolated PBMCs were stored in 10% DMSO at −80°C.

T cells and B cells present in macaque PBMCs were separated using anti-CD3-Alexa fluor 700 (non-human primate, Miltenyi Biotec) and PE-Cy7 anti-CD19 (BioLegend, clone H1B19) Abs in a BD FACSAreiaIII cell sorter. Recombinant FHbp (28) with His-tag was conjugated to Alexa-488 NHS ester (Pierce) based on amine coupling following the manufacturer’s instructions and purified using a Ni-NTA agarose column, and Alexa-488 conjugated FHbp was validated for its binding with human FH before using as bait in flow cytometry (data not shown). Among the B-cell population, anti-mutant FHbp antibody-producing B cells were detected with FHbp mutant conjugated to Alexa-488 and the positive B cells were then sorted as single cells into a FrameStar^®^ Break-A-Way PCR plate containing 5 μL of lysis buffer (mentioned below) compatible with downstream cDNA synthesis PCR. Ninety-six-well plates containing single sorted B cells were centrifuged at 4°C and then stored frozen at −80°C for later use.

**Table:** Sorting/Lysis buffer.

Components	10 mL buffer volume
RT buffer 10×	1,000 μL
dNTP (10 mM)	700 μL
0.5% NP-40 (10% stock)	500 μL
10 mM DTT (0.1 M stock)	1,000 μL
100 µL of RNase out (400 U/µL)	100 μL
Water	6,700 μL

### Synthesis of heavy- and light-chain antibody fragment cDNA

Frozen PCR plates with sorted single B cells were thawed on ice. A High-Capacity cDNA Reverse Transcription Kit (Thermo Fisher Scientific) was used for cDNA synthesis for RT buffer 10× and other reagent components, as suggested by the manufacturer with minor modifications as described below.

As a minor modification, instead of the random primers supplied in the kit, CH2 domain-specific reverse primers for VH outer (5′ CCA CGC ATG TGA CCT CAG GG 3′), Kappa outer (5′ TGA GGT GAA AGA TGA GCT CGA G 3′), and Lambda outer (5′ TCC CCT GGG ATC CTG CAG 3′), designed for macaque Fabs (**Table 1**), were used at a final concentration of 0.2 μM in 10 µL. Five microliters of a mix of all three primers was added to each well of the 96-well plates containing flow cytometry-sorted single B-cell lysates in 5 µL of PCR compatible lysis buffer. The primer-mixed sample plate was sealed and used for cDNA synthesis following the cDNA synthesis protocol mentioned below:

**Table:** cDNA synthesis mix.

Component	For 10 µL reaction vol
10× RT buffer	
dNTP mix (100 mM)	0.2 µL
All three primers	0.2 μM each
RT enzyme	
Carrier DNA	
Water Make up to	5 µL

**Table 1:** Primers used in cDNA synthesis form sorted single B cells.

Targeted region	CH2 domain-specific reverse primers for cDNA synthesis
VH outer	5′ CCA CGC ATG TGA CCT CAG GG 3′
Kappa outer	5′ TGA GGT GAA AGA TGA GCT CGA G 3′
Lambda outer	5′ TCC CCT GGG ATC CTG CAG 3′

**Table:** cDNA synthesis/PCR setup

Time	Temperature
10 min	25°C
120 min	37°C
5 min	85°C
Hold	4°C

The first PCR amplification involved the generation of a heavy and light chain of Fab fragments. Synthesized cDNA from each well was used to amplify heavy chain and light chain (Kappa or Lambda) of Fab. From each well containing 10 µL of cDNA (5 µL lysis buffer + 5 µL cDNA synthesis mix), 3 μL was used for VH amplification, 3 μL was used for Kappa amplification, and 3 µL was used for Lambda amplification. For the first PCR of heavy chain, CH1 reverse (5′ CTCGAGTTCCCCCTCCCTCGCTGGCCTCTCACCAACTCTCTTGTCCACCTTGGTGTTGCT 3′) and VH outer forward primers (**Table 2**) at a final concentration of 0.08 µM in 25 µL total reaction volume were used. The first sets of primers for light chains were Kappa reverse outer and Lambda reverse outer along with Kappa forward outer and Lambda forward outer, respectively, and were at final concentrations of 0.08 µM in 25 µL total volume (**Table 2**).

**Table 2:** List of all primers used for the first PCR.

Antibody region	Reverse primer
CH1	5′ CTCGAGTTCCCCCTCCCTCGCTGGCCTCTCACCAACTCTCTTGTCCACCTTGGTGTTGCT 3′

VH family	Forward primers (outer primers)
VH1	5′ TGCCCAGCCGGCGATGGCCCAGGTGCAGCTGGTGCAG 3′
VH2	5′ TGCCCAGCCGGCGATGGCCCAGGTGACCTTGAAGGAGTCT 3′
VH 3A	5′ TGCCCAGCCGGCGATGGCCGAGGTGCAGCTGGTGGAG 3′
VH 3B	5′ TGCCCAGCCGGCGATGGCCGTGGTGCAGCTGGTGGAG 3′
VH4	5′ TGCCCAGCCGGCGATGGCCCAGCTGCAGCTGCAGGAG 3′
VH5	5′ TGCCCAGCCGGCGATGGCCGAGGTGCAGCTGGTGCAG 3′
VH6	5′ TGCCCAGCCGGCGATGGCCCAGGTGCAGCTGCAGGAG 3′
VH7	5′ TGCCCAGCCGGCGATGGCCCAGGTGCAGCTGGTGCAG 3′

Kappa family	Kappa outer forward primers, first PCR
VK1.L1	5′ GGCTCCTKCTGCTCTGGCTC 3′
VK2.L1	5′ ATGARGYTCCCTGCTCAG 3′
VK3.L1	5′ ATGGAARCCCCAGCWCAGC 3′
VK4.L1	5′ ATGGTGTCACAGACCCAAGTC 3′
VK5.L1	5′ ATGGCATCCCAGGTTCASC 3′
VK6A.L1	5′ ATGTTGTCTCCATCACAACTC 3′
VK6B.L1	5′ ATGGTGTCCCCATTGCAACTC 3′
VK7.L1	5′ ATGGGGTCCTGGGCTCC 3′
**Outer reverse**	5′ TGAGGTGAAAGATGAGCTCGAG 3′

Lambda family	Lambda outer forward primers, first PCR
VL1.L1	5′ ATGGCCTGGTYYCCTCTC 3′
VL2.L1	5′ ATGGCCTGGRCTCTGCTCC 3′
VL3A.L1	5′ ATGGCCTGGATTCCTCTC 3′
VL3B.L1	5′ ATGGCCTGGACCTTTCTC 3′
VL3C.L1	5′ ATGGCCTGGACCCCTCCC 3′
VL4A.L1	5′ ATGGCCTGGGTCTCCTTC 3′
VL4B.L1	5′ ATGGCCTGGACCCCACTC 3′
VL5.L1	5′ ATGGCCTGGACTCCTCTC 3′
VL6.L1	5′ ATGGCCTGGGCTCCACTCC 3′
VL8.L1	5′ ATGGCCTGGATGATGCTTC 3′
VL9.L1	5′ ATGGCCTGGGCTCCTCTG 3′
Outer reverse	5′ TCCCCTGGGATCCTGCAG 3′

All PCRs were carried out in a total volume of 25 µL using the Taq-polymerase kit from NEB. Initial denaturation was carried out for 3 min for hot start, and the next 15-s denaturation was carried out with 1-min annealing and a final extension of 10 min for 25 cycles for VH and 30 cycles for VL, as summarized below.

### Nested PCR

For the second round of nested PCR, 2.5 µL of the first PCR product was used as a template, and a second set of primers, VH reverse inner and VH forward inner, were used at a final concentration of 1 µM in 25 µL total reaction volume for VH amplification. For light-chain amplification, Kappa inner forward and Kappa inner reverse primers at final concentrations of 0.4 µM in 25 µL were used for Kappa chain amplification. The same strategy was used for Lambda chain amplification as well. **Table 3** summarizes all primers used in nested PCR. The PCR reactions were similar to the first PCR setup except for changes in annealing temperature, as summarized below.

**Table:** First PCR.

Temperature	Time	Cycles
95	3 min	
95	15 s	25 VH, 30 VL
61 VH, 50 VL	1 min	25 VH, 30 VL
72	10 min	
4	hold	

**Table 3:** List of primers used for nested (second) PCR amplifications. The same VH forward primers were used as outer forward primers for the first PCR.

VH family	Inner forward primers
VH1	5′ TGCCCAGCCGGCGATGGCCCAGGTGCAGCTGGTGCAG 3′
VH2	5′ TGCCCAGCCGGCGATGGCCCAGGTGACCTTGAAGGAGTCT 3′
VH 3A	5′ TGCCCAGCCGGCGATGGCCGAGGTGCAGCTGGTGGAG 3′
VH 3B	5′ TGCCCAGCCGGCGATGGCCGTGGTGCAGCTGGTGGAG 3′
VH4	5′ TGCCCAGCCGGCGATGGCCCAGCTGCAGCTGCAGGAG 3′
VH5	5′ TGCCCAGCCGGCGATGGCCGAGGTGCAGCTGGTGCAG 3′
VH6	5′ TGCCCAGCCGGCGATGGCCCAGGTGCAGCTGCAGGAG 3′
VH7	5′ TGCCCAGCCGGCGATGGCCCAGGTGCAGCTGGTGCAG 3′
VH inner reverse	5′ ATATATGGCCACGATGGCCCCTTGGTGGAGGCTGAGGA 3′

Kappa Family	Inner forward primers
IGKV1A	5′ ATATAT CACAAAGTGGACATCCAGATGACCCAGTCTC 3′
IGKV1B	5′ ATATAT CACAAAGTGGATTTCCAGATGATTCAGTCTC 3′
IGKV1C	5′ATATAT CACAAAGTGGACATCCAGATGTCCCAGTCTC 3′
IGKV2A	5′ ATATAT CACAAAGTG GATGTTGTGATGACTCAG 3′
IGKV2B	5′ ATATAT CACAAAGTG GATGTTGCGATGACTCAG 3′
IGKV2D	5′ ATATAT CACAAAGTG GATATTGTGATGACCCAG 3′
IGKV2E	5′ ATATAT CACAAAGTG GATACTGTGATGACCCAG 3′
IGKV3A	5′ ATATAT CACAAAGTG GAAATTGTAATGACGCAGTCT 3′
IGKV3B	5′ ATATAT CACAAAGTG CAAGTTATATTGACACAGTCT 3′
IGKV4A	5′ ATATAT CACAAAGTG GATATCAGGCTGACCCAG 3′
IGKV4B	5′ ATATAT CACAAAGTG GACATTGTGATGACCCAG 3′
IGKV5A	5′ ATATAT CACAAAGTG GAAACGATACTCACACAG 3′
IGKV5B	5′ ATATAT CACAAAGTG GAAATGATACTCACACAG 3′
IGKV6A	5′ ATATAT CACAAAGTG GAAATTGTGCTGACTCAG 3′
IGKV6B	5′ ATATAT CACAAAGTG GATATTGTGATGACTCAG 3′
Kappa inner reverse	5′ GGCCATCGCCGGCTGGGCAGCGAGGAGCAGCAGACCAGCAGCAGCGGTCGGCAGCAGGTATTTCATATGTATATCTCCTTCTAACACTCTCCTCTGTTGAAGCT 3′

λ Family	Inner forward primers
IGLV1A	5′ ATATATCACAAAGTGCAGTCTGTACTGACTCAGCTGC 3′
IGLV1B	5′ ATATATCACAAAGTGCAGTCTGTGCTGACTCAGCCAC 3′
IGLV1C	5′ ATATATCACAAAGTGCAGGCAGGGCTGACTCAGCCAC 3′
IGLV2A	5′ ATATATCACAAAGTGCAGTCTGCCCCGATTCAGTCT 3′
IGLV2B	5′ ATATATCACAAAGTGCAGGCTGCCCTGACTCAGCCT 3′
IGLV2C	5′ ATATATCACAAAGTGCAGGCTGCCCCGACTCAGTCT 3′
IGLV2D	5′ ATATATCACAAAGTGCAGTCTGCCCCGACTCAGCCT 3′
IGLV3A	5′ ATATATCACAAAGTGTCCTCTGAGCTGACTCAG 3′
IGLV3B	5′ ATATATCACAAAGTGTCCTCTGGGCTGACTCAG 3′
IGLV3C	5′ ATATATCACAAAGTGTCCTCTGAGCTGGCTCAG 3′
IGLV3D	5′ ATATATCACAAAGTGTCCTCTCAGCTGACTCAG 3′
IGLV3D	5′ ATATATCACAAAGTGTCCTATGAGCTGACACAG 3′
IGLV3E	5′ ATATATCACAAAGTGCAGCCTGTGCTGACTCAGTCG 3′
IGLV4A	5′ ATATATCACAAAGTGCTGTCTGTGCTGACTCAGCCC 3′
IGLV4C	5′ ATATATCACAAAGTGCAGTCTTGCGCTGACTCA 3′
IGLV5A	5′ ATATATCACAAAGTGCAGTCTGTGCTGACTCA 3′
IGLV5B	5′ ATATATCACAAAGTGCAGCCTGTGCTGACTCA 3′
IGLV5C	5′ ATATATCACAAAGTGAAGCCTATGCTGACTCA 3′
λ inner reverse	5′ GGCCATCGCCGGCTGGGCAGCGAGGAGCAGCAGACCAGCAGCAGCGGTCGGCAGCAGGTATTTCATATGTATATCTCCTTCTATGAACATTCTGCAGGGGCCAC 3′

**Table:** 

Temperature	Time	Cycles
95	3 min	
95	15 s	25 VH, 30 VL
64 VH, 67 VL	1 min	25 VH, 30 VL
72	10 min	
4	hold	

All nested PCR reactions were carried out in total volumes of 25 µL using a Taq polymerase kit from NEB. Five microliters of the nested PCR products, mixed with 1× SYBR™ Green, was loaded onto a 1% agarose gel to check for amplification. For either monkey 120 (WT FHbp) or monkey 184 (mutant FHbp), the original B-cell-sorted and cDNA-synthesized PCR plate was tracked and continued until the nested PCR product from each well was analyzed on agarose gel. To minimize the workload, first, all VH products were documented on agarose gel, and only the corresponding positive wells were backtracked and selected for running the Kappa and Lambda light chains.

### Linkage PCR for native pairing of heavy and light chains

Positive heavy-chain and the corresponding light-chain products from respective wells were used as templates for linkage and amplification of Fabs. For linkage, 5′ of heavy chain and 3′ of light chain were designed to have overlapping bases of 40 bp length that came from the PelB sequence of *E. coli.* In a 25-µL linkage reaction mix, 5 µL of 1-in-10,000 diluted nested PCR products from heavy- and light-chain reactions was mixed with NEB Taq polymerase components without primers. After linkage PCR, 0.08 µM forward nested primers for light chain and 0.08 µM reverse nested primers (**Table 3**) for heavy chain were added to the reaction mix and subjected to PCR amplification of linked Fab. Linkage PCR was set up using the conditions described in the table below.

**Table:** Linkage PCR setup—step 1, without primer.

Temperature	Time	Cycles
95	3 min	
95	30 s	15
65	1 min	15
72	1 min	15
72	5 min	
4	hold	

**Table:** Linkage PCR setup—step 2, post primer addition.

Temperature	Time	Cycles
95	1 min	
63	1.5 min	25
68	2 min	25
68	1 min	

### Gel extraction and purification of linked Fab

Extra care was taken to keep track of the wells selected for corresponding positive heavy and light chains to ensure native pairing. The successful native pairing and linkage PCR product was analyzed based on its size on an agarose gel. After agarose gel verification of VH–VL for either VH-Kappa or VH-Lambda, the natively paired linked Fabs from 7 to 10 different wells were pooled in one group, to keep the number of Fab under 10 and make the downstream process easier, mixed with 1× SYBR™ Green (Thermo Fisher Scientific), and ran on 1% agarose gel. Under UV illumination, 1,100-bp bands corresponding to linked products were cut and purified using the gel extraction kit from Qiagen. Bound DNA was eluted in 25 µL of H_2_O, and the concentration was measured in a nanodrop.

### Cloning of Fab fragments into pGEMT

The pGEMT easy kit from Promega was used for cloning Fabs into the pGEMT vector. As all the amplifications were performed using Taq polymerase, the PCR products were poly A tailed, thus facilitating ligation with the pGEMT vector. Ligated DNA was transformed into *E. coli* DH5-alpha cells (NEB) as per by the manufacturer’s instructions. After 1 h incubation at 37°C with shaking in 1 mL of LB (Luria–Bertani) broth (Lennox, Invitrogen), 100 µL of transformed cells was then plated on an LB agar plate with 50 µg/mL ampicillin and incubated overnight at 37°C. The rest of the cells (transformed with pooled linked DNA) were added to 5 mL of LB media with 50 µg/mL ampicillin and grown overnight at 37°C in a shaker incubator.

### Screening of Fab-positive colonies by PCR

Colonies from Fab-pGEM-T transformed *E. coli* DH5-alpha cells were tested for the presence of Fab inserts by colony PCR. From each transformation plate, 10 to 15 colonies were picked and resuspended in 200 µL of LB media. This step was included to confirm ligation and transformation of the linked product as a downstream checkpoint for gene Fab confirmation. Five microliters of the prepared colony mixture was used as a template in a PCR reaction set up with 0.08 µM VH reverse and a mix of all Kappa or Lambda forward primers. The rest of the protocol was the same as the linkage PCR step 2. PCR products mixed with 1× SYBR™ Green were then loaded on 1% agarose gel to verify the insertion of Fab in pGEMT.

### Isolation of mix Fab DNA from pGEM-T clones

Bacteria were harvested from the liquid cultures of the Fab-positive colony pools from the PCR screen and plasmid DNA was isolated (Qiagen QIAprep Spin Miniprep Kit). Isolated and purified mixed pGEM-T-Fab DNA and modified pET22a expression vector were double digested with restriction enzymes, HindIII and SfiI. To this end, the plasmids were first digested with HindIII HF at 37°C for 2 h in 1× cut smart buffer from NEB, and then SfiI was added to the same reaction mix and incubated at 50°C for 3 h to make a second cut. The digested DNA product was run on agarose gel, extracted, and purified. Gel-extracted 1,100-bp Fab DNA was ligated to the already digested pET22a vector at 4°C overnight, and the ligated product was transformed into DH5-alpha cells. The transformed colonies were screened by colony PCR using a mix of Kappa or Lambda forward and VH reverses primers, and the Fab containing pET22a plasmid was isolated from a 5-mL liquid culture. DNA isolated from mixed cultures that were positive for Fab insertions were saved for the transformation of *E. coli* BL-21 cells.

### Fab screening by colony blot

Because of the large number of colonies and as part of a continuous screening process for Fabs, the colonies on the blot were not numbered. However, after careful matching, post-selection of all positive colonies for further screening, as a blind strategy, numbering was randomly but in continuity opted for the respective WT or mutant FHbp-immunized samples. *E. coli* BL-21 (DE3) pLysE cells were transformed with the pET plasmid containing Fab fragments (plasmid extracted and verified by sequencing) and spread onto LB agar plates containing 50 µg/mL ampicillin and incubated overnight at 37°C. In a different Petri plate, half of the plate was carefully spread with the 1A12 (human Fab against FHbp) pET clone as a positive control, while the CH1 vector was spread to the other half of the plate as a negative control. The next day, grown colonies were transferred onto a nitrocellulose membrane, and the membrane was carefully placed on a fresh LB agar plate containing 50 µg/mL ampicillin and 0.2 mM IPTG. Colony positions were marked on the membrane using a needle for the accurate selection of colonies post-colony blot. The master plate and plate with membrane-spotted colonies were incubated again at 37°C, with the master plate being incubated overnight, whereas the replica plate with membrane was incubated only for 4 h. After incubation for 4 h, the membrane was carefully taken out of the plate with forceps to proceed with colony blotting. The colony-bearing membrane was processed according to the QIAexpressionist colony blot protocol. Briefly, at room temperature, the membrane was denatured first in 10% sodium dodecyl sulfate (SDS) for 10 min and then in 0.5 M NaOH for 5 min, followed by incubation in neutralizing solution (0.5 M Tris-HCl and 1.5 M NaCl, pH 7.4) for 5 min, and the final incubation was in SSC (saline-sodium citrate) buffer for 15 min. Membrane was then washed thrice in PBS (phosphate-buffered saline) and incubated with HRP (horseradish peroxidase) conjugated primary antibody against Fab (anti-macaque, Ig H+L, R&D) at 4°C overnight. The next day, after three washes with PBS, the membrane was developed with TMB (tetramethylbenzidine) substrate (Bio-Rad). This procedure produces a visible purple insoluble precipitate when oxidized by HRP conjugated to anti-Fab. After drying, the developed blot was photographed with a simple camera. It is important to consider that the colony blot process is only a qualitative screening process, but it has the potential for high throughput and is an optional or interchangeable step with enzyme-linked immunosorbent assay (ELISA).

### Fab screening by ELISA

Colonies that were positive for Fab expression on the colony blot screen were picked from the master plate and inoculated into 2 mL of LB broth with 3% ethanol and grown at 37°C in a shaking incubator. After 5 h, 0.1 mM IPTG was directly added to the culture without checking the optical density (OD), and shaking was continued for approximately 18 to 20 h. The next day, expression cultures were harvested, and cell pellets were lysed with 0.1% lysozyme in Tris-HCl buffer. The double-diluted lysates were added to a 96-well ELISA plate already coated with recombinantly expressed 2 µg/mL of purified WT FHbp (24). To detect all positive Fabs, only the WT FHbp was used for coating. After incubation for 2 h, the plate was blocked with 2.5% milk in PBS for 1 h. After one wash with PBS containing 0.1% tween 20 (PBST), the plate was probed for Fab expression with HRP-conjugated anti-macaque Fab (R&D) diluted in PBST for 1 h. Then, TMB substrate was added and the intensity of substrate color was measured in an ELISA plate reader.

### Standardization of Fab expression

Fab expression was optimized before pursuing large-scale purification by scrutinizing for ethanol versus glucose in combination with LB versus super broth. Fab-expressing BL21 (DE3) pLysE cells were tested for Fab expression in two different media, namely, LB and super broth, with either 1% glucose or 3% ethanol and induced with varying concentrations of IPTG (0.1, 0.2, and 0.5 mM). Cell lysates from 2 mL of induced cultures were analyzed on Bis-Tris PAGE (polyacrylamide gel electrophoresis) gel and immunoblots. The same procedure was followed for immunoblotting as performed with colony blot post-denaturation and membrane wash with PBS. Heat-denatured samples (95°C for 5 min) were also tested for disulfide bond reduction in Fabs with 2% BME (β-mercaptoethanol) before loading on NuPAGE 4%–12% Bis-Tris protein gel (Invitrogen).

### Fab expression, purification, and characterization

All the recombinant WT and mutant FHbp proteins used in this study have already been characterized for their functions (29–31). Positive clones from the ELISA screen were grown and expressed in large 2-L cultures for the downstream purification process. Briefly, multiple colonies (10–20) of each positive clone were picked and inoculated into 50 mL of LB broth containing 50 µg/mL ampicillin and 3% of ethanol and grown overnight for 17–18 h at 37°C with shaking at 250 rpm. Picking multiple colonies of a single positive clone ensures average to better growth and expression, as the chances of picking a single low-growing colony due to phenotypic heterogeneity (32) are counterbalanced by other better-expressing colonies from the same clone. The next day, 2 L of LB broth containing 50 µg/mL ampicillin and 3% ethanol was inoculated with 2% of the overnight culture and grown for 4–5 h at 37°C till OD 600 reached approximately 0.8. At the desired OD, the culture was induced with 1 mM IPTG stock for a final concentration of 0.1 mM IPTG and grown overnight at 30°C for approximately 17–18 h. The bacterial cells were harvested from the induced cultures by centrifugation at 4,000 rpm. Subsequently, the periplasmic fraction from the cell pellet was separated by the lysis of cells in binding buffer (20 mM Tris-HCl and 150 mM NaCl, pH 8.0) containing 0.1% lysozyme and incubating on ice for 1 h, followed by three freeze–thaw cycles using liquid nitrogen. The resulting cell lysate was centrifuged at 19,000 rpm for 15 min to remove cell debris, and the supernatant was carefully collected. Fab was purified from the lysate by binding it to a protein G column (GE Healthcare) and eluting it with 100 mM glycine, pH 2.0, on an AKTA protein purification system (Cytiva Life Sciences). Protein G is highly suitable for the purification of both Lambda and Kappa Fabs. Before elution and for neutralization, 200 µL/well of Tris-HCl, pH 9.0, was added to the fraction collector for 1 mL of fraction collection. Fractions from the AKTA peak were heat denatured in a 2× Laemmli SDS-PAGE sample buffer (Bio-Rad) and run on Bis-Tris PAGE gel, followed by staining in Coomassie brilliant blue (Bio-Rad). Visually pure fractions were pooled and dialyzed against PBS and then concentrated using 10-kDa-cutoff spin columns (Millipore).

Purified Fabs were then again assessed for their functional ability to bind FHbp in an ELISA experiment similar to that mentioned in the earlier method section for screening of Fabs. For the purified Fabs ELISA experiment, human Fab 7B10 generated commercially and expressed in the lab was used as a positive control, and the anti-macaque Ab recognizes it (cross-reacts with human Ab).

## Results

### Single B-cell sorting

**Figure 1:**
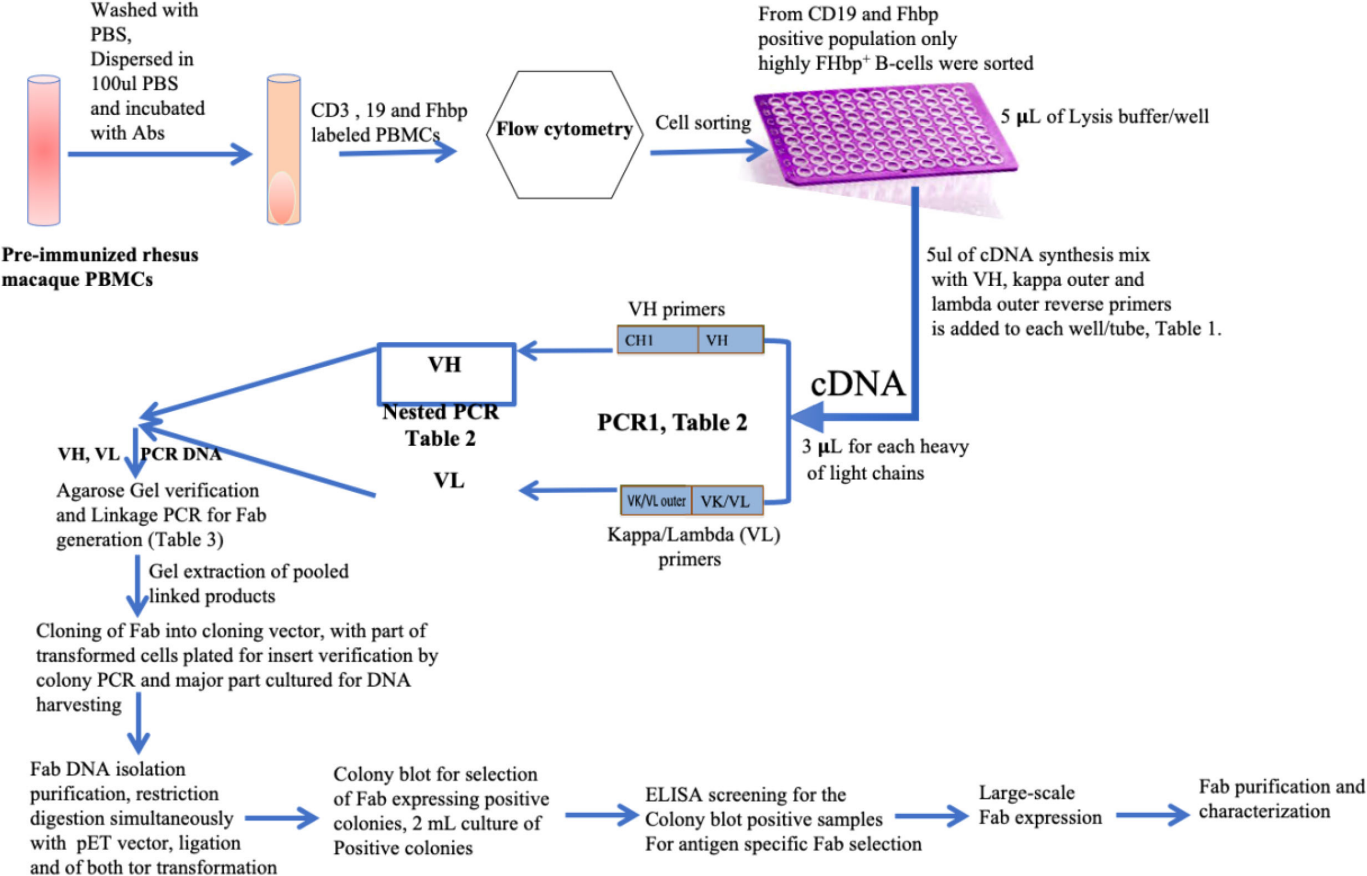
Schematic presentation of the workflow depicting steps needed in the Fab generation.

**Figure 2:**
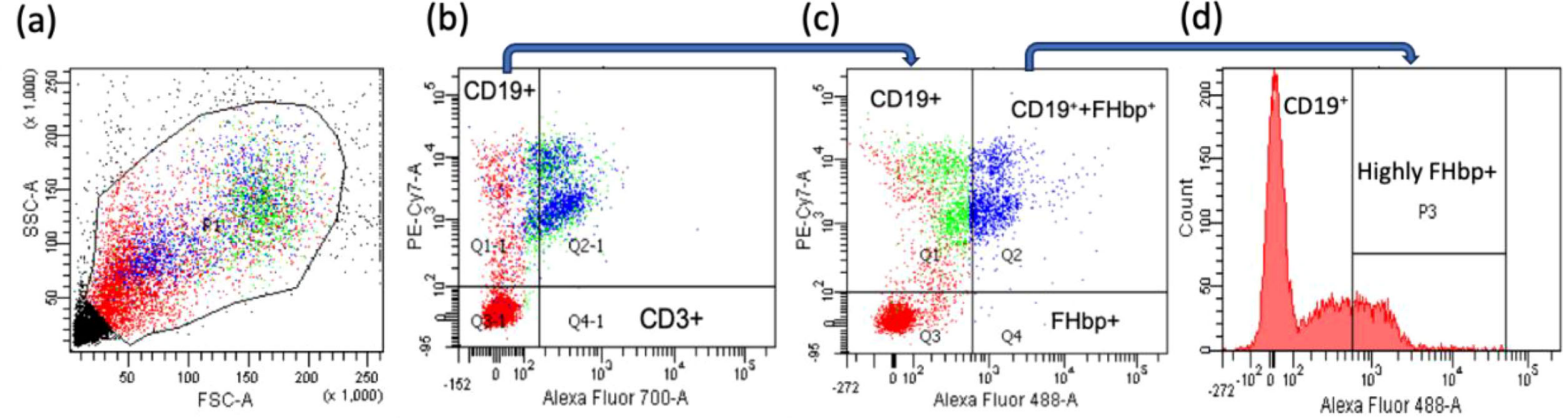
Representative image showing the strategy for flow cytometry-based single B-cell sorting to select anti-FHbp antibody-secreting B cells from mutant FHbp-immunized macaque. **(a)** Monkey PBMCs scatter plot showing colors for the cell population being analyzed under different gating strategies, **(b)** CD3^+^ cells for B cell negative and CD19^+^ for B cell positive, **(c)** FHbp-positive B cells within all B-cell population, and **(d)** CD19 and FHbp double-positive cells were selected for the highly FHbp-positive population of B cells (P3) from the histogram plot for sorting. Arrows above the panels indicate sequential gating.

The steps employed in the Fab generation are summarized as a schematic presentation in **Figure 1**. The strategy used for sorting FHbp-positive single B cells is shown in **Figure 2**. As depicted in **Figure 2a**, a multistep gating strategy was employed to select single B cells expressing an antibody against FHbp on its surface using Alexa Fluor-488-conjugated mutant FHbp as bait.

### Agarose gel verification of VH and VL nested PCR products generated from macaque B cells

**Figure 3:**
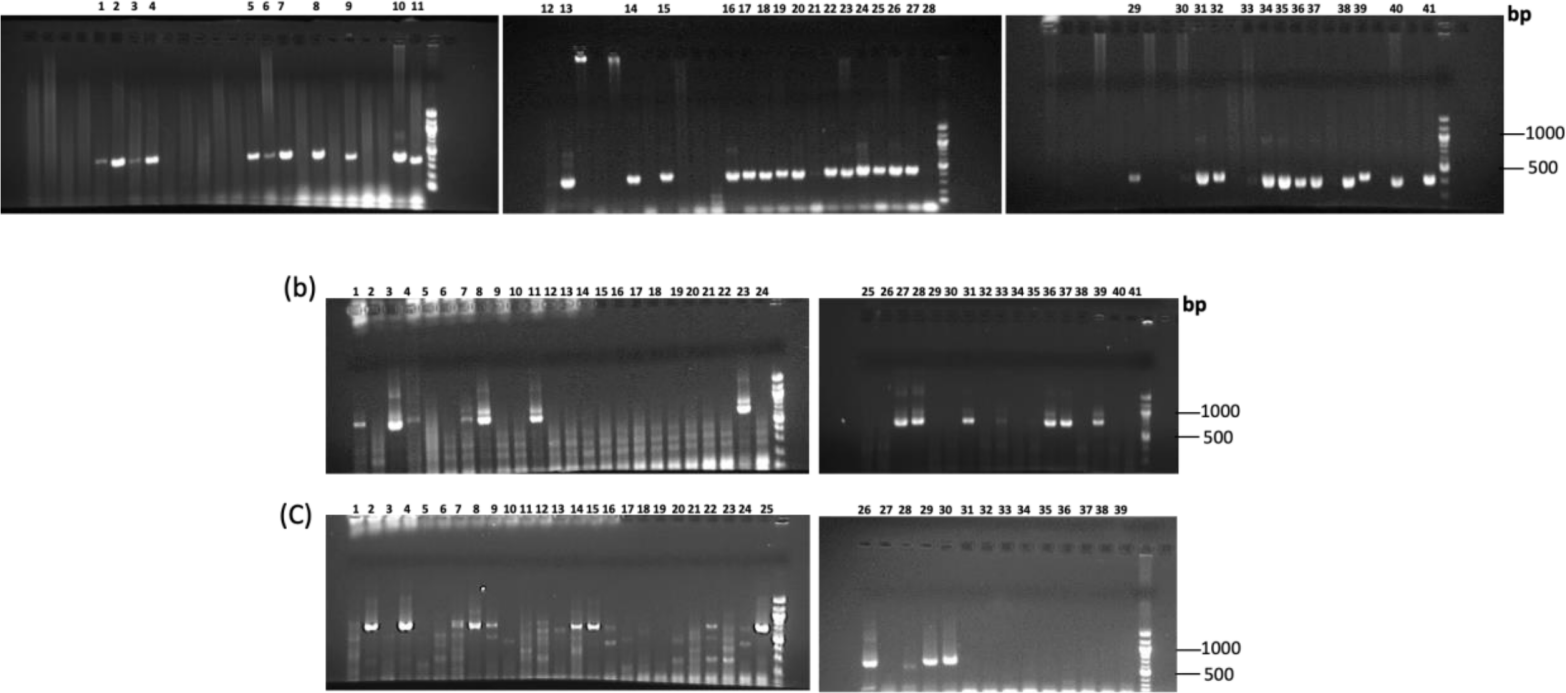
Agarose gel analysis of PCR-amplified heavy- and light-chain DNA from single B cells of macaques immunized with mutant FHbp. Shown above are the images of agarose gels with the PCR products of heavy and light chains amplified from B-cell DNA of macaque number 184, with heavy chains (VH) seen as 400-bp bands **(a)**, Kappa light chains seen as 700-bp bands **(b)**, and Lambda light chains seen as 700-bp bands **(c)**. Only VH-positive samples were tested for light chains; hence, lanes 1–41 in **(b)** and lanes 1–39 in **(c)** correspond to the positive lanes of VH in **(a)**.

Agarose gel verification of VH and VL nested PCR products were performed by running the samples on agarose gel to visually analyze for the presence of amplified VH and VL (**Figure 3a**). Results are shown for amplified products from macaque B cells, which were injected with modified (mutant) FHbp. The PCR products of 400 bp correspond to amplified VH fragments, as shown in **Figure 3**. A macaque injected with a mutant version of FHbp yielded approximately 56% VH-positive sorted cells, out of 72 samples analyzed from the 96-well B-cell sorted plate. Only positive VH cDNA were amplified for Kappa and Lambda. As shown in **Figures 3b, c**, with 700-bp bands, the yield for both Kappa and Lambda are similar at approximately 10%.

**Figure 4:**
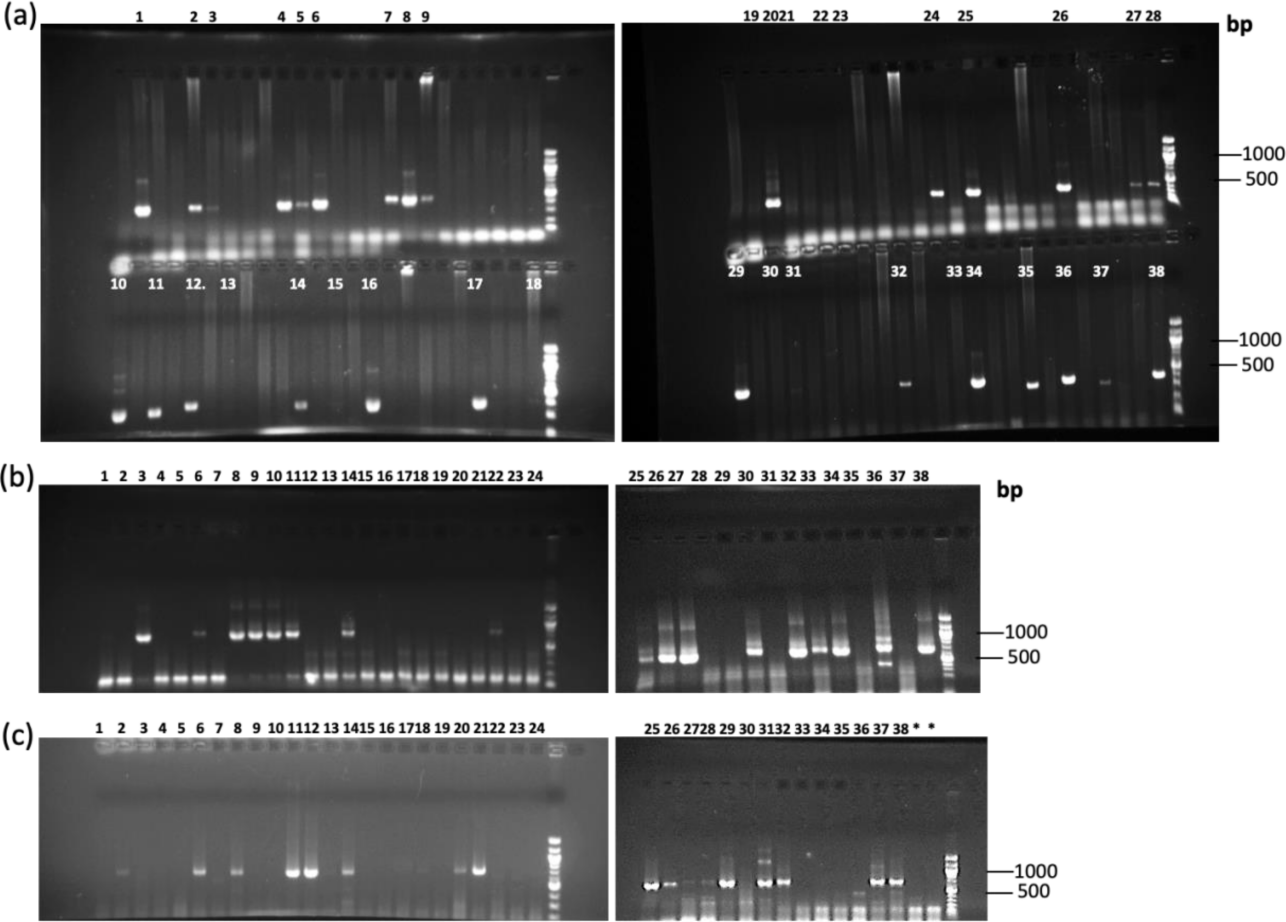
Agarose gel analysis of PCR-amplified heavy- and light-chain DNA from single B cells of macaques immunized with wild-type FHbp. Shown above are the images of agarose gels with PCR products of heavy and light chains amplified from B-cell DNA of macaque number 120, with heavy chains (VL) seen as 400-bp bands **(a)**, Kappa light chains seen as 400-bp bands **(b)**, and Lambda light chains seen as 700-bp bands **(c)**. Only VH-positive samples (lanes numbered) were tested for light-chain amplification. Thus, **(b, c)** lanes correspond to the positive lanes of VH in **(a)**. The “*” indicates water samples that serve as negative control.

Agarose gel validation for VH from the WT FHbp challenged macaque PBMCs is shown in **Figure 4a**. As calculated for mutant samples, the WT DNA data shown indicate a total of approximately 39% yield for VH amplification, as only 38 samples turned VH positive out of 96 samples from the 96-well B-cell sorted plate. From the positive VH cDNA samples, again the ratio of amplified Kappa and Lambda products was equal (**Figures 4b, c**).

The positive VH and VL products are required for Fab generation. To develop a functional Fab, a native pairing of VH and VL is essential, and thus, both chains must be from the same B cell (same well), which was ensured by linking VH and VL from the same well by accurate tracking back of the corresponding wells.

### Analysis of VH and VL linked products to generate Fabs

**Figure 5:**
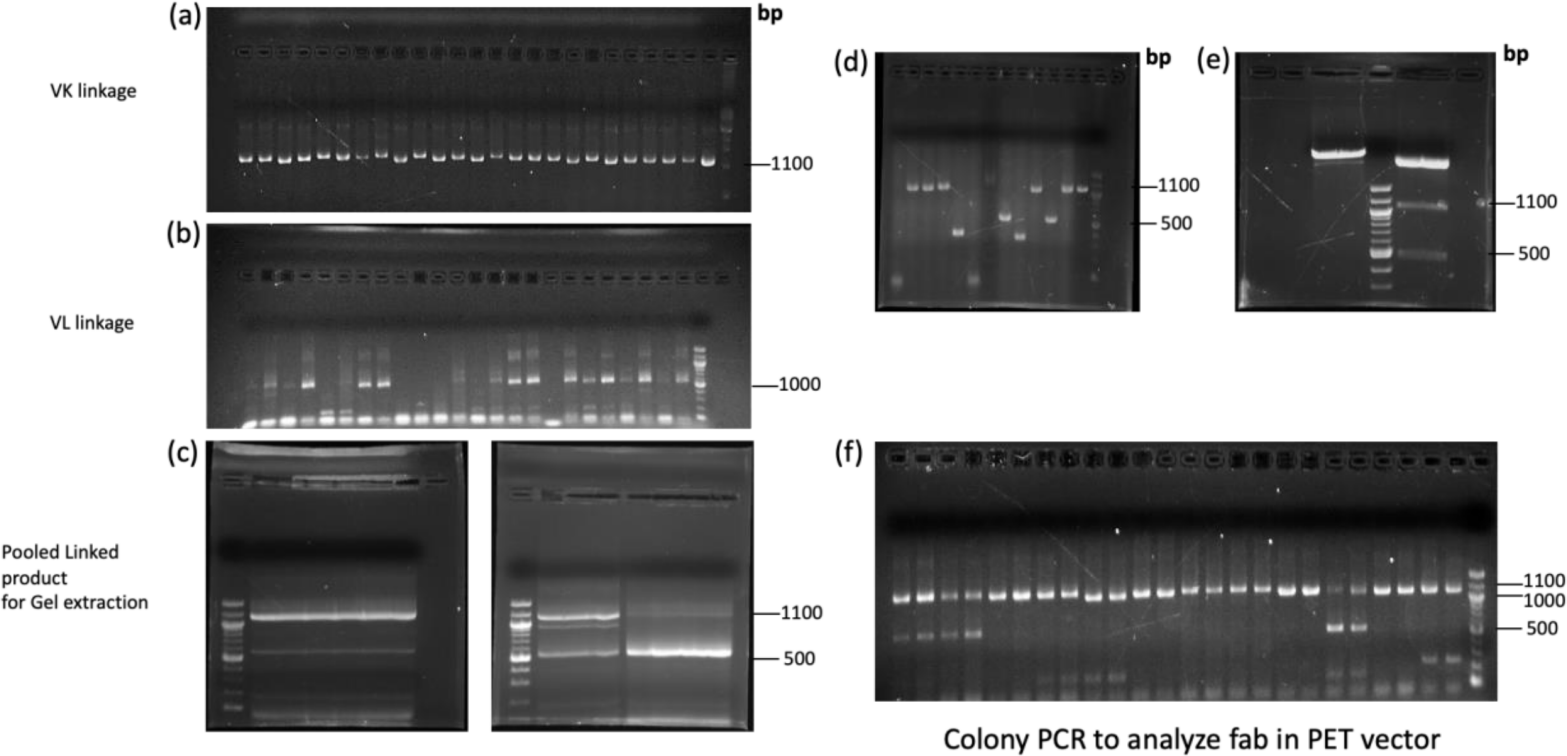
Agarose gel analysis of linkage PCR products. **(a)** Fabs in the form of VH+Kappa linkage product with a size of 1,100 bp. **(b)** VH+Lambda linkage product with a size of 1,100 bp. **(c)** Pooled VH+Kappa (left gel) and 2 different pools for VH+Lambda for gel extraction. **(d)** Colony PCR product after purified Fab cloned into pGEMT. **(e)** Restriction digestion of pET vector (left of the ladder), and to the right of the ladder is pGEMT with Fab DNA insertion. **(f)** Colony PCR confirmation of Fab in the pET 22a vector. A similar process was carried out for VH+Kappa and VH+Lambda. For representation, only VH+Kappa is shown in **(d, e)**.

The success of a linkage experiment can be confirmed either by sequencing or simply by analyzing the DNA on agarose gel for the correct and expected product size. Given the large number of samples, the best rationale is to run the linked products on an agarose gel and examine the size of the DNA. To this end, VH and VL primers were designed to amplify products with overlapping ends as represented in **Figure 5**. Unpurified linkage products were analyzed on agarose gel for a 1,100 (400 VH + 700 VL)-bp PCR product and can be seen in **Figure 5a** for VH+Kappa and **Figure 3b** for VH+Lambda. The linkage amplification of VH and Kappa always yielded better and cleaner products compared to VH and Lambda linkage, with 25 vs. 17 out of 25 VH+VL linked clones (wells) analyzed after PCR amplification.

Five to seven individual linked products from VH-Kappa and VH-Lambda were pooled separately within the group to create a mixed product pool for screening. Pooled samples were then gel-extracted and purified (**Figure 5c**). Lambda linkage samples were grouped based on product specificity of the PCR products, i.e., better products into one group and another group had individuals with some unspecific products included (**Figure 5c**, right gel, second lane from the ladder).

### Cloning and validation of Fab inserts

To obtain a functional Fab, the linked product must be cloned into a desired vector, and it is crucial for the products to be clean for obtaining a better cloning outcome. We purified mixed linkage but natively paired products (Fabs) and cloned them into pGEMT. Since all the colonies will not have the Fab insert in them and thus to further consolidate the working samples, colonies grown overnight were screened for positive insertion of Fabs by colony PCR as visualized on agarose gel (**Figure 5d**) with 1,100 bp for VH+VL. Fifty percent of the picked colonies for PCR showed positive insertion of Fab into the pGEMT. The isolated DNA from the Fab inserted clones were restriction digested, and the 1,100-bp band was gel-extracted, as shown in **Figure 5e**. The pET22a vector digestion was also examined in the same agarose gel. It is evident from the DNA gel documentation that the digested pET vector runs at the expected lower speed (high molecular weight), while the digested pGEMT runs at a higher speed (low molecular weight) with the release of the Fab insert. Again, to confirm that the pET22a vector was carrying our Fab insert for expression, colony PCR was performed, and a 1,100-bp band was visualized on agarose gel (**Figure 5f**).

### Colony blot screening of functional Fabs

**Figure 6:**
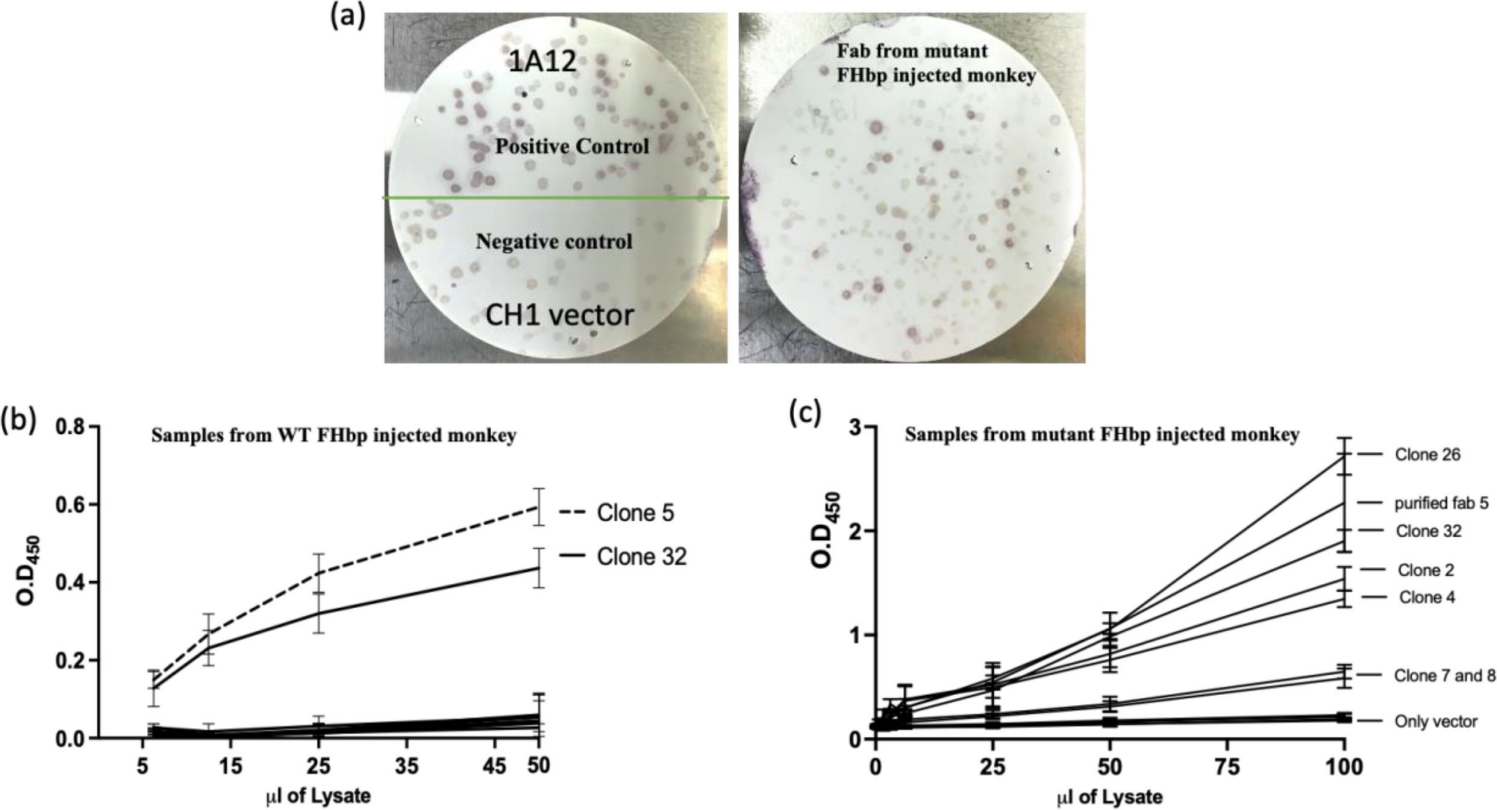
Fab protein expression screening by colony blot and ELISA. **(a)** Left blot shows positive control Fab 1A12 and negative control CH1 vector, with a green line separating both. The right blot represents one of the blots for Fabs derived from B cells of a macaque immunized with mutant FHbp. **(b)** Expression of Fabs in *E. coli* lysates by ELISA (cloned from macaque immunized with wild-type FHbp). **(c)** ELISA-based fab expression screening of positive colonies on colony blot for Fabs generated from B cells of a macaque (No. 184) injected with mutant FHbp. In **(c)**, the purified Fab clone 5 from **(b)** was used as a positive control. Also, Fab 1, 6, and 10 from macaque 184 are at the bottom of the ELISA curve.

After validation of the Fab harboring pET22a clones, the setup will be ready for streamlining the expression of Fabs. As a quick and easy way to select the Fab expression-positive colonies from the culture plates, all colonies were lifted onto PVDF membranes and checked for Fab-positive colonies by comparing with positive and negative controls. BL21 (DE3) pLyse cells transformed with the human Fab 1A12 expressing plasmid or the pET22a vector only served as a positive and negative control, respectively. As presented in **Figure 6a**, the left blot shows negative and positive controls, and the right blot shows the Fab sample tested. 1A12, as a positive control, exhibited the brightest dots compared to the CH1 vector as a negative control, while the Fab sample plate revealed a mix of positive and negative signals. The colony blot provides visual confirmation of whether colonies are positive or not for protein expression. However, it does not inform whether the positive colony is expressing our Fab of interest. Thus, to identify which colony is Fab expressing among the colony blot positive colonies, ELISA is the most straightforward and cost-effective method.

### ELISA screening for colony blot positive clones

A total of 30 strongly positive colonies with bright signals on the colony blot screen were cultured again, and only the 10 best-growing cultures from both WT and mutant FHbp injected macaques were tested by ELISA for Fab expression before proceeding with large-scale expression and purification. To minimize the workload of handling a large number of samples, and as an extra measure, to enhance the selection likelihood of highly expressing clones, the pre-culture step was skipped for ELISA screening of the Fab-expressing colonies. Two sets of triplicate data for the detection of Fab against FHbp by ELISA are presented in **Figures 6b, c**. Both **Figures 6b, c** are results of different sets of experiments, with **Figure 6b** showing all Fabs from WT FHbp, while **Figure 6c** exhibits Fabs from mutant FHbp-immunized monkey PBMCs. The cell lysate-based ELISA shown here has varying binding affinity for the generated Fab with FHbp. Usually, Ab expression in bacterial systems is low-yielding, so we decided to standardize the expression level before going for large-scale expression and purification.

### Fab expression in the presence of ethanol

**Figure 7:**
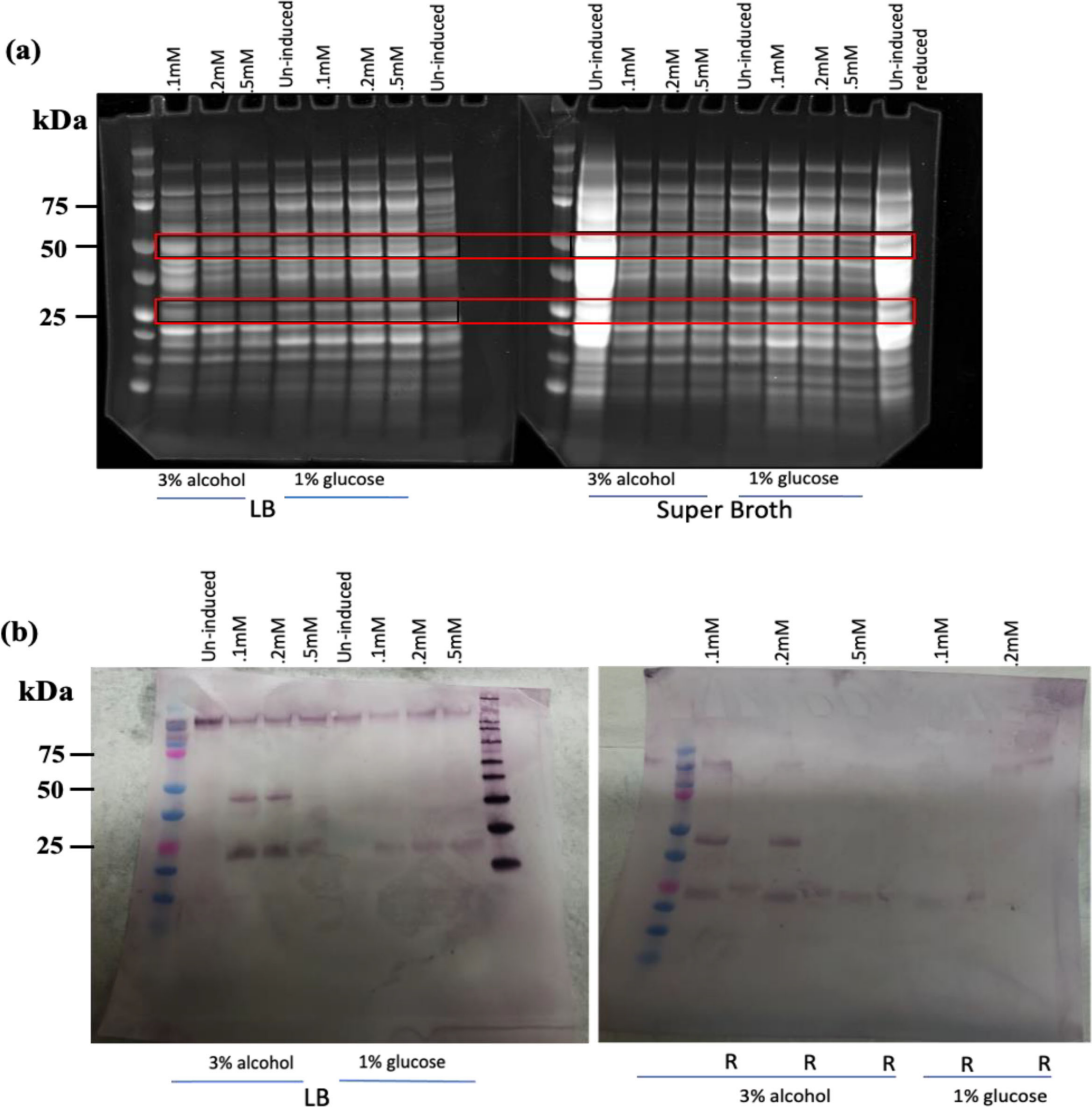
Bis-Tris and Western blot analysis of Fab protein expression strategy. **(a)** Left protein gel shows Fab expression in LB, whereas the right gel shows Fab expression in super broth, in the presence of alcohol or glucose and with varying concentrations of IPTG as indicated above the lanes. The antibody chains are marked with two black boxes across the gels, one approximately 50 kDa on top and other approximately 25 kDa toward the bottom of the non-reduced denatured gel. **(b)** Left blot: Fab expressed in LB in the presence of alcohol or glucose, run non-reduced and denatured and immunoblotted. The right blot contains the same samples with reduced samples included (R).

Different media with various concentrations of IPTG in the presence of either glucose or ethanol were tested for maximal Fab expression. Results indicate that the Macaque Fab was better expressed in LB with 3% ethanol (v/v) when induced with 0.1 mM IPTG. In **Figure 7a**, boxed 50- and 25-kDa bands in LB with 3% ethanol and 0.1 mM IPTG are most evident with a visible gradient from 0.1 to 0.5 mM. Expression in super broth appears to be more non-specific. Fabs are known to resolve at approximately 50 and 25 kDa in non-reduced conditions, and 50 kDa is reduced to 25 kDa in reducing sample buffer, making all Fab chains 25 kDa (33), and our data reveal the same, as depicted in the **Figure 7b**. Immunoblotting (**Figure 7b**, left blot) of the same samples used in Bis-Tris PAGE supports the notion of better Fab expression in LB with 3% ethanol and 0.1 mM IPTG. Additionally, Fabs are well reduced, specifically those expressed in LB ethanol as can be seen on immune blotting (**Figure 6b**, right blot). The control Fabs (1A12 in colony blot or 7B10 in ELISA) were not expressed and tested for alcohol use, but with the methods used for expression, these Fabs were only sufficiently expressed for some experiments, as pointed out in the relevant published study, and CH1 is the vector alone without insert (34).

### Large-scale Fab expression and purification

**Figure 8:**
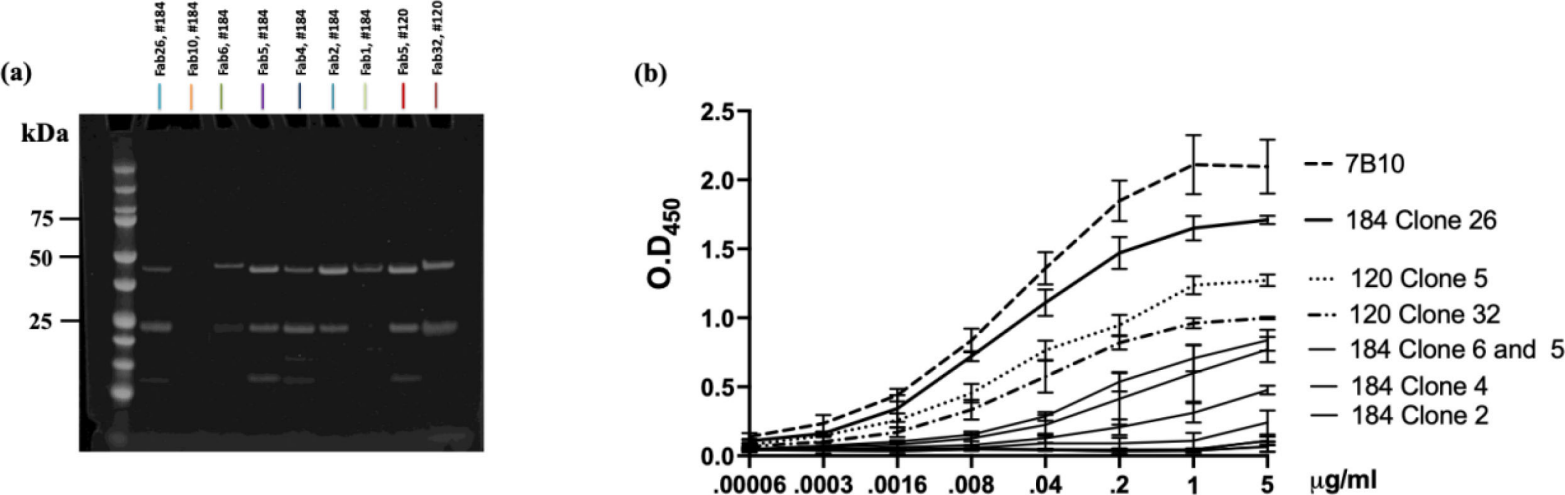
Evaluation of purified Fabs by ELISA and SDS-PAGE. **(a)** SDS-PAGE analysis of all Fabs expressed and purified by FPLC. The names of the Fab clone and macaque number are indicated above the lanes. **(b)** ELISA study for all the purified Fabs to analyze the binding specificity to FHbp wherein the ELISA plate was coated with 2 μg/mL of FHbp followed by the addition of double dilutions of each Fab and probing with anti-macaque Fab. In the ELISA experiment, purified human anti-FHbp Fab 7B10 was used as a positive control. Fab 1 and 6 from Macaque 184 are at the bottom of the binding curves and not labeled in the image. Fab 10 from macaque 184 was completely degraded and thus not visible in the gel and was not included in the final ELISA test.

FHbp-specific Fabs identified by ELISA screen were individually expressed in large LB culture with 3% ethanol and 0.1 mM IPTG as inducer. **Figure 8a** shows the purified Fabs run on Bis-Tris PAGE gel and stained with Coomassie blue. Bands smaller than 25 kDa observed for some purified Fabs could indicate degradation due to multiple freeze–thaw cycles, while for monkey 184 #1, only 50 kDa was observed even in repeated gel runs, likely due to non-separation of intact heavy and light chains. Factors for these observed anomalies were not part of the study, but these observations point to the non-identical stability of Fabs. All the purified macaque Fabs were characterized by ELISA for their binding to FHbp (**Figure 8b**).

## Discussion

In the current study, we have developed a systematic, cost-effective, and streamlined method for generating Fabs from single B cells sorted out of PBMCs from rhesus macaques immunized with *N. meningitidis* FHbp (24). The first critical and challenging step for generating Fabs is targeting antigen-specific single B cells for sorting (35). To increase the specificity of single B-cell sorting for a specific antigen by flow cytometry, we used our in-house FHbp conjugated to Alexa-488 as a bait to fish out B cells presenting anti-FHbp Abs on their surface. Similar to previously published work on single B-cell sorting, we also used CD3 and CD19 markers (36–38) to separate T cells from B cells, but also employed Alexa-488-FHbp double mutant with mutations R41S and H248L (25) for selecting positive B cells against FHbp. Compared to earlier studies (36–38), our strategy of using the immunogen as bait for B-cell sorting minimizes false-positive selection by relying only on pan CD3 and CD19, and Ab-based selection also enhances the total yield. Also, for cost-effectiveness yet with high fidelity, a set of human Abs can be replaced with the conjugated antigen for sorting B cells.

Our VH amplification data from **Figures 3**, **4** showed 41 vs. 39 out of 72 (56%) and 96 (39%) samples analyzed on agarose gel, respectively, for macaques immunized with mutant FHbp or WT FHbp, indicating a higher likelihood of isolating anti-FHbp Ab-producing B cells when mutant FHbp is used as bait. This could reflect very low binding or interference by FH with mutant FHbp, which may improve the immunization process, though this needs further study. In this milieu, one similar study using a panel of Abs targeting B cells reported five positive heavy-chain amplifications out of 15 samples loaded on agarose gel (40%) (39), which is comparable to our VH amplification yield from WT-immunized macaque PBMCs. Another study on mAb generation from immunized mice and rabbits using a cocktail of Abs revealed a maximum of 40% antigen specific B cells sorting (40). Thus, our findings support the use of a conjugated antigen as a crucial parameter for selecting a single B cell expressing the desired antibody. Accordingly, the cocktail of Abs used for B-cell sorting can be replaced by an optimized and well-characterized conjugated antigen against the expected antibody. Although the sample size is very small, with only two macaques (one immunized with WT FHbp and the other injected with mutant FHbp), the data also indicate that mutant FHbp may be better at generating memory B cells, perhaps because it has lower affinity for human FH (25), thereby increasing the overall availability of the immunogen. As such, these comparisons are descriptive, as only one macaque per immunogen was evaluated. The present study supports the notion of further experimental validation of antigen-specific B-cell production, where the antigen can be engineered to alleviate non-specific or undesired interactions in immunized animals (41), resulting in a lower antigen dose requirement. Furthermore, using a modified antigen bait for sorting specific Ab-secreting B cells is a boon for reducing the non-specificity of single B-cell screening.

As pointed out in the Introduction, most of the earlier studies are not complete from generation to characterization of Fabs (36, 39, 42), and unlike the mentioned studies, our amplification of light chain from the VH-positive cDNA was evenly favored for both Kappa and Lambda, as depicted in **Figures 2a**, **3a**. Careful matching of Kappa and Lambda chain PCR products for each sample used revealed that the maximum number of positive VH samples were from a single cell as there were either Kappa or Lambda in corresponding lanes (**Figures 3b, c**, **4b, c**). The study by Jian et al. reported a recovery of 27% for Kappa and 25% for Lambda, and a final recovery of 25% for light-chain and heavy-chain pairing, while Tiller et al. documented (agarose gel) a difference of 50% in the amplification of Kappa and Lambda chains. Distinct from previous Fab development studies, our methodology is focused on first validating the VH-positive wells from the original plate and then performing VL amplification only for positive and corresponding wells, aiming to save both time and resources.

In all our linkage experiments, the 3′ overlapping region in Kappa products seemed to snap better with the 5′ overlapping region in VH, compared to Lambda products, as evidenced by the agarose gel analysis of linked products (**Figures 4a, b**). It is also reflected in **Figure 4c**, which shows that only one lane was used for the VH+Kappa linkage product, while two lanes were dedicated to the VH+Lambda linkage product, as the Lambda linkage product is not as clean as Kappa. Not part of this study, but the observed disparity could be partially explained by the differences in the physicochemical properties of the Kappa and **L**ambda chains in their CDR3 regions (43). A very high dilution of 1:10,000 was finally used, and this could be because the linkage step is a continuous third PCR step using unpurified PCR products, leading to continuous accumulation of PCR-inhibitory by-products. In addition, gel extraction of pooled linked PCR products (**Figure 5c**) resulted in much cleaner DNA, and the relatively impure PCR products could be separated from non-specific amplifications. Henceforth, the downstream cloning process with clean PCR amplicons became more reliable with better yield as shown in **Figure 4d**. The cleaning and purification of DNA products provides an opportunity for direct sequencing if desired. Additionally, gel extraction and purification of DNA are needed for efficient downstream processing (44).

Because of the large number of Fab-positive pGEMT colonies, isolation of Fab DNA from individual colony by restriction digestion would be a time-intensive process. Instead, Fab containing pGEMT DNAs were extracted from pooled colonies, and then restriction digestion was performed. Simultaneous digestion of the pET vector to be used and pGEMT with the inserted Fab clone with the same restriction enzyme set and gel extraction (**Figure 5e**) again helps to achieve better transformation for cloning the Fab fragment in the pET vector. This is supported by 100% of colonies picked for colony PCR showing a 1,100-bp band for Fab insertions in the pET vector (**Figure 5f**), which were further transformed into *E. coli* BL21(DE3) pLysE cells. To further explain, a colony blot was chosen as a desired step because of its ease at identifying and picking only a Fab-expressing colony. As a downstream process, colony blot further consolidates the number of positive Fabs, an old but time-efficient and cost-effective procedure (45). As shown in **Figure 5a** Fab-expressing individual colonies chosen by the colony blot screen were picked and grown in liquid media to induce Fab expression. Putative Fab-expressing colonies were analyzed by ELISA to determine their binding to FHbp. We found Fabs with different degrees of binding specificity to their antigens. Even though the ELISA results shown in **Figures 6b, c** are based on Fabs expressed in cell lysate, they identified several good candidate Fabs for large-scale expression and purification. Because the colony blot experiment involves the denaturation of samples, it is fundamentally different from ELISA, where samples are used in a non-denatured state. Thus, using colony blot minimizes the undermining of any Fab-positive sample by selecting all antigen-independent Fab-expressing colonies, while ELISA detects only native and functional protein, antigen-specific or non-specific (46). The colony blot and the membrane post SDS-PAGE run for Fabs under non-reduced and reduced conditions are cost-effective steps for screening and validating Fabs without the use of any high-end instrument, as they were developed using TMB substrate (Bio-Rad) and HRP conjugated to anti-Fab for a visible precipitate and imaged by a simple camera.

Knowing Fab/Ab expression in *E. coli* is not always optimal (47, 48); we tried different combinations of media and components to achieve a better yield. Our data signify that supplementing LB medium with 3% ethanol resulted in a superior Fab yield compared to standard LB or super broth supplemented with 3% ethanol (**Figures 7a, b**). The addition of alcohol has been demonstrated previously to play a role in indole acetic acid production, which reduces the stress level in bacteria (49). Only one previous study has described the potential use of alcohol for protein production in *E. coli* (27), but the authors did not show how a combination with different concentrations of IPTG can influence the yield in *E. coli*. Additionally, we have validated our results by immunoblotting to authenticate Fabs (**Figure 7b**). The higher molecular weights are most likely the protein aggregate, including possible heteropolymerization, as the samples were unpurified cell lysates (50, 51), which is not seen in the reduced condition. As evident in the left and right panels of **Figure 7b**, 3% alcohol provided better yield in comparison to no alcohol in LB media, and based on the expression profile of Fabs in **Figure 7a** compared to LB media, super broth was ruled out for large-scale Fab expression. Our purified Fab ELISA results (**Figure 8b**) for binding to FHbp support the efficiency of the procedure from Fab screening to Fab purification and functional characterization. Of the 10 purified Fabs, only clone 26 from the mutant FHbp-immunized monkey is comparable to 7B10. Though clones 5 and 32 from the WT FHbp-immunized monkey and clones 6 and 5 from the mutant FHbp-immunized monkey showed moderate binding, further study is needed for better characterization. The positive control is known for its affinity to FHbp and has been extensively characterized (52). From the ELISA screening, even very low-binding or non-binding Fabs were included for expression, purification, and characterization to ensure consistency in results obtained from linked steps and to rule out the idea that purified Fabs behave differently. Importantly, in agreement with better VH amplification, the ELISA data with purified Fab also point to the mutant antigen (FHbp) generating better Abs. Out of nine purified Fabs, six were isolated from the macaques injected with the double mutant FHbp. Even though the specificity is debatable, a greater number of different Fabs produced by the mutant antigen (FHbp) is apparent from the data.

Taken together, we have established a reproducible, less expensive, antigen-specific, simplified, and high-yielding methodology for Fab generation and its expression in *E. coli*. We have also successfully demonstrated the positive manipulatory effect of 3% ethanol on the expression of Fab in bacterial systems. The alcohol data are more qualitative than quantitative, but the observed difference is obvious. Thus, our results indicate that LB media with added ethanol may improve the expression of select antibody fragments in *E. coli*. In addition, our data also hint that the Abs isolated from mutant FHbp-immunized macaques were better and greater in number than the WT antigen-induced Abs. However, these findings require further substantiation.

Because of time and resource constraints, the study has its limitations in the characterization of Fabs and functional assays (inhibitory). As part of a larger project, this study aimed to establish the working protocol and purify Fabs. Further detailed functional/inhibitory characterization of the purified Fabs and analysis of the repertoire polarization are desired.

## Data Availability

The datasets analyzed for this study can be found in the GenBank: Accession numbers: PZ497125, PZ497126, PZ497127, PZ497128, PZ497129, PZ497130, PZ497131, PZ497132, PZ497133, PZ497134, PZ497135, PZ497136, PZ497137 and PZ497138.
